# 5′-methylcytosine and 5′-hydroxymethylcytosine Each Provide Epigenetic Information to the Mouse Zygote

**DOI:** 10.1371/journal.pone.0063689

**Published:** 2013-05-14

**Authors:** Yan Li, Chris O’Neill

**Affiliations:** Centre for Developmental and Regenerative Medicine, Kolling Institute for Medical Research, Sydney Medical School, University of Sydney, Sydney, New South Wales, Australia; Goethe University, Germany

## Abstract

Covalent modification of cytosine nucleotides within the genome encode essential epigenetic information, with methylation (5meC) and hydroxymethylation (5hmC) having received most attention. It has been proposed that the formation of 5hmC is an intermediate in the active demethylation of 5meC. Some reports show that global loss of 5meC in the newly fertilised embryo is accompanied by increased 5hmC, but others have failed to confirm this finding. These analyses have relied on immuno-localization of these modifications. In this study we have established the conditions required for equilibrium binding of antibodies to 5meC and 5hmC in zygotes. Simultaneous detection of these antigens required denaturation of chromatin by acid treatment followed by antigen retrieval by tryptic digestion. Equilibrium binding then required incubation at 4°C for greater than 6 h. These are more demanding conditions than generally reported and resulted in the consistent detection of 5meC and 5hmC in both male and female pronuclei throughout zygotic maturation. No dynamic reciprocal change in the level of 5meC relative to 5hmC was observed. Both 5meC and 5hmC accumulated within the peri-nucleolar regions and this was more pronounced in the male pronucleus. Staining of 5meC was relatively more intense within the cortical and 5hmC in the central regions of pronuclei. The results are not consistent with a role for 5hmC in global demethylation in the zygote. The persistence of both modifications throughout zygotic maturation, and their differing patterns of localization and solvent exposure infer each modification provides its own epigenetic information to the early embryo.

## Introduction

Lineage specific patterns of gene expression rely upon mitotically heritable epigenetic modifications to the genome. One important epigenetic mechanism is the covalent modification (methylation) of cytosine within CpG dinucleotides. Hypermethylation of regions of the genome are associated with the parent-of-origin dependent mono-allelic silencing of imprinted loci, silencing of potentially dangerous genetic elements (including endogenous retrotransposons), and X-chromosome inactivation (in females) [1], [2], [3]. The level of DNA methylation of a loci is correlated with the level of chromatin accessibility and the binding of cofactors such as P300 (a histone acetyltransferase) [4]. These functions, and the mitotic heritability of methylation patterns, implicate this modification as an important component of the cell’s lineage specific epigenetic landscape. Reprogramming of this pattern between lineages requires a mechanism of remodelling the methylation status of the genome. A key component of this process is a mechanism for selective removal of methylation, yet no definitive evidence for the identity of an active mammalian demethylase currently exists.

A longstanding paradigm of epigenetic reprogramming involves the remodelling of the nucleus to the totipotent state that is considered to occur in the early embryo soon after fertilisation. It is argued that immediately following mammalian fertilisation there is global active demethylation of the paternally-derived genome relative to the maternally-derived genome [5], [6]. This model holds that demethylation occurs prior to the first round of DNA replication and is followed by further progressive passive demethylation over subsequent cell-cycles. This round of putative active demethylation in the zygote has become the dominant model for screening and identifying potential demethylases and is therefore of broad significance.

A number of possible mechanisms for this active demethylation have been advanced [7], [8], [9] yet to date none have found wide experimental support [10]. Recently, the family of ten-eleven translocation dioxygenases (TET) were found to catalyse the oxidation of 5′-methylcytosine into a range of metabolites, including 5′-hydroxymethylcytosine (5hmC) [11]. 5hmC is widely distributed among tissues, including pluripotent stem cells [11], [12]. It appears to be a favourable substrate for deamination by enzymes, including activation-induced deaminase [13], thus a role for 5hmC as an intermediate in a demethylation pathway has been proposed [14]. TET3 was detected within the paternally-derived (male) pronucleus and some studies found 5meC and 5hmC had a reciprocal pattern of immunolocalization during zygote maturation. Staining of 5meC was lost and 5hmC accumulated within the male but not the maternally-derived (female) pronucleus [15], [16]. This pattern was not obvious in *Tet^−/−^* zygotes [15]. In contrast to these findings, another study [17] did not detect this reciprocal pattern of expression of 5meC and 5hmC staining during zygotic maturation. High levels of staining of 5hmC in both the male and female pronuclei were observed but 5meC was enriched only in the female pronucleus. These conflicting reports on the dynamics of 5meC and 5hmC during zygotic maturation cloud our understanding of the processes of epigenetic reprogramming in the zygote and require resolution.

Only small amounts of DNA can be recovered from the early embryo so much of the experimental support for the asymmetric demethylation of the male pronucleus is based on immunolocalization of the 5meC antigen within zygotes. There are many reports of a progressive loss of 5meC staining from the male but not female pronucleus [18], [19], [20]. Yet, a recent analysis [21] showed that this apparent loss of methylation was accounted for by a progressive onset of acid-resistant masking of the 5meC epitope in the zygote during its maturation. When the staining procedure was modified to achieve full antigenic retrieval by tryptic digestion, the 5meC antigen was found to persist in both the male and female pronuclei throughout zygotic maturation and also over the cleavage stage of development [21]. This observation is consistent with recent chemical analysis of methylation sites (by reduced representation bisulfite sequencing [22]) which showed broadly similar levels of methylation in the male and female pronuclei. Another study using this methodology showed that methylation levels in the early embryo were higher than could be accounted for by the current model of post-fertilisation demethylation [23]. These analyses, however, used the bisulfite conversion sequencing technique, which is now recognised to not distinguish between 5meC and 5hmC (and other metabolites) [24]. Thus, bisulfite sequencing analyses do not exclude the possibility of reciprocal changes in the levels of the 5meC and 5hmC in the pronuclei.

Immunolocalization is a critical tool in distinguishing between the various cytosine modifications. There are no chemical means currently available for genome-wide simultaneous analysis of 5meC and 5hmC at the base level in the small amounts of DNA that can be feasibly recovered from the zygote. Given the conflicting results reported for immunolocalization analyses of these cytosine modifications [15], [17], and the inability of bisulfite sequencing to distinguish between the metabolites [24], this study aimed to define the requirements for reliable antigenic retrieval and simultaneous detection of the 5mC and 5hmC and to reassess the levels and patterns of hmC and 5meC staining during zygotic maturation. This reanalysis did not find evidence consistent with a role for 5hmC in global demethylation in the zygote. The study found different patterns of localization of these two modifications within the pronuclei and differing levels of solvent exposure. The results indicate differing epigenetic roles for these two cytosine modifications in the early embryo.

## Results

Comparative immunolocalization requires that the antibody-antigen interaction approaches thermodynamic equilibrium. Important variables that can affect this equilibrium include the time and temperature of incubation with antibodies, and the level of solvent exposure of the antigen. The study used antibodies that have been well validated for their specificity and at concentrations that optimised the signal to noise output of each primary antibody relative to its non-immune control. The optimal incubation time and temperature required for saturable staining to be achieved were then assessed for each antigen. PN5 stage zygotes were fixed, blocked and subjected to the published antigen retrieval techniques for each antigen (acid treatment for 5hmC [17] and acid plus trypsin treatment for 5meC [21]) and then incubated with antibodies at room temperature or 4°C for periods from 1 to 18 h. Equatorial optical sections were performed through each pronuclei. The captured images were subjected to image analysis to assess relative changes in the average staining intensity/pixel and total levels of staining across the equatorial cross-section of each pronuclei.

**Figure 1:**
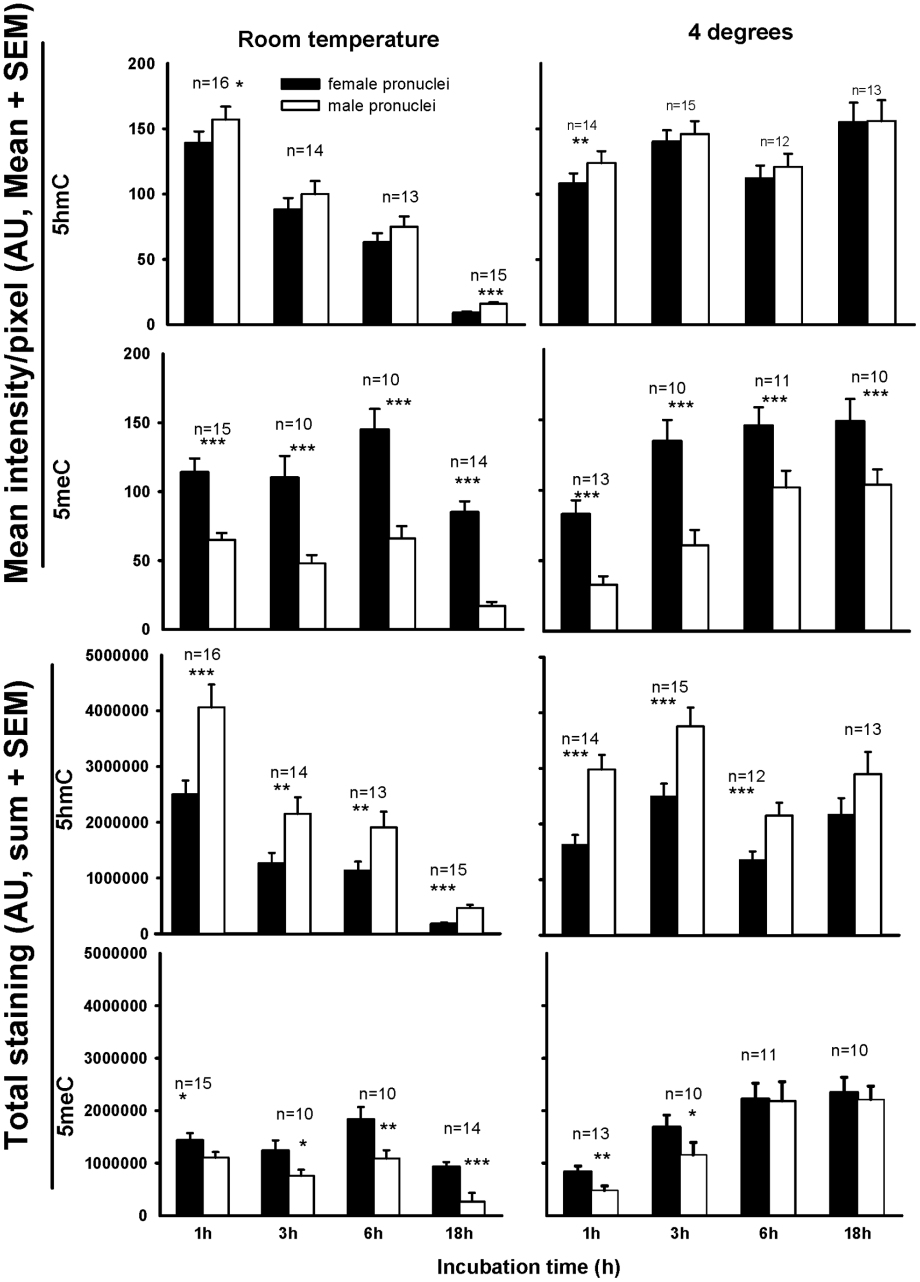
The influence of primary antibody incubation time and temperature on the level of staining of the 5hmC and 5meC antigens in the male and female pronuclei of mouse PN5 stage zygotes. Zygotes were collected directly from the oviduct, fixed and prepared for staining after either acid pretreatment (5hmC or acid plus trypsin pretreatment (5meC). Zygotes were incubated with primary antibodies for between 1 and 18 h at either room temperature or 4°C. Staining levels in the male and female pronuclei of each zygote was assessed as the optical density of staining in each pronuclei. The data is shown as the mean intensity/pixel within each pronuclei (top panel) and the total staining of all pixels within each pronuclei (bottom panel). The data is expressed as the SEM of arbitrary units of optical density measured (AU). The number of zygotes measure for each treatment (n = ) is shown above each bar. The zygotes were collected from three independent replicates. Differences in staining levels at individual times were assessed by paired T-tests, *P<0.05; **P<0.01; and ***P<0.001. The changes in staining levels relative to time were assessed by univariate analysis using the general linear model and significant effects are noted within the results text. In each case conditions for staining and imaging were managed so that no signal was detected from the non-immune negative control treatments, and these same conditions were used for imaging primary antibody staining.

Staining of 5meC and 5hmC were observed in both pronuclei after incubation for 1 h at room temperature. Incubation for longer periods at room temperature, however, resulted in a progressive reduction in both the intensity and total staining of both 5meC and 5hmC (Fig. 1). The intensity of 5hmC staining was higher (p<0.01), and 5mC lower (p<0.001) in the male pronuclei across these incubation periods after staining at room temperature. By contrast, after incubation at 4°C, 5hmC staining remained relatively stable over 18 h, while 5meC staining showed a progressive and significant increase in staining with incubation time (p<0.01), reaching a plateau from 6 h. After incubation at 4°C, total 5hmC staining was still consistently higher in the male pronucleus over shorter incubation times but after 18 h incubations this difference was no longer significant (p>0.05). By contrast, 5meC staining intensity/pixel was lower in the male pronucleus across each incubation time. Total 5meC staining was lower in the male after short periods of incubation with antibody, but under equilibrium conditions of prolonged incubation (>6 h) this difference was no longer apparent. This difference in kinetics of binding shows that the thermodynamics of binding to each of these antigens differed, and both differed between each pronuclei. Saturable binding to both antigens could be achieved after the exposure to antibodies for greater than 6 h at 4°C.

Subjective analysis of this staining is susceptible to an easy optical illusion. The staining of 5meC in the male pronucleus appeared less than the female. This is because the male pronucleus is significantly larger (571.4±21.2 µm^2^, mean ± SEM equatorial cross-sectional area for fixed and denatured zygotes) than the female (389.4±14.5) (P<0.001). Thus, while the staining intensity/pixel was lower in the male than female, the larger size of the male meant that the overall level of staining was not different (P>0.05, Fig. 1).

**Figure 2:**
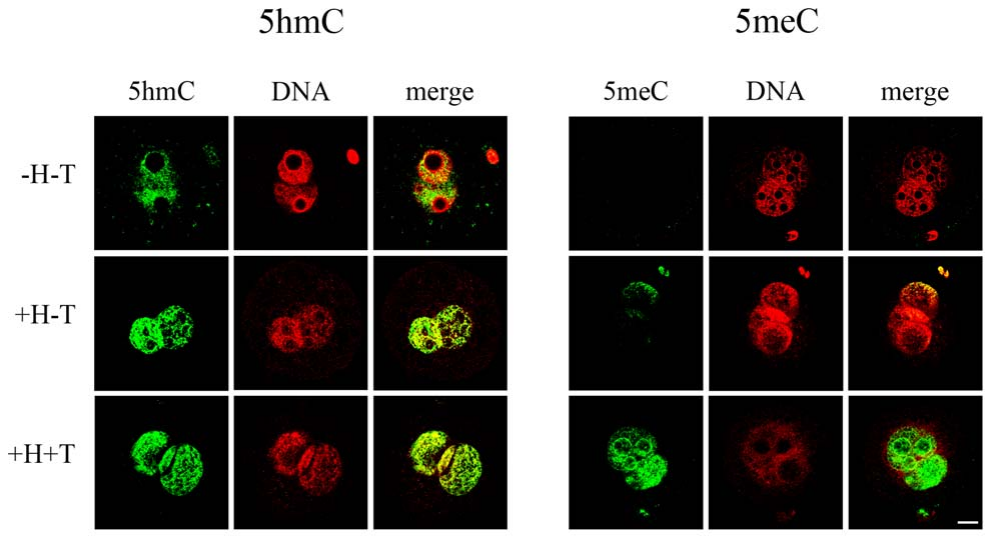
The effect of antigen retrieval procedures on the detection of 5hmC and 5meC in the mouse PN5 stage zygote. Zygotes were collected directly from the oviduct and fixed and prepared for staining. After antigen retrieval steps, zygotes were incubated with primary antibodies for 1 h at room temperature (5hmC) or 18 h at 4°C (5meC) and each primary antibody was detected by an FITC-labeled secondary antibody. The pronuclei were counter-stained for DNA with PI and the FITC and DNA images merged. Three epitope retrieval approaches were under taken: −H-T, zygotes were not pretreated with either acid (HCI) or trypsin; +H-T, they were pretreated with acid only; and +H+T, epitope retrieval was by a combination of acid and trypsin treatment. The images are representative of three independent replicates. In each case conditions for staining and imaging were managed so that no signal was detected from the non-immune negative control treatments, and these same conditions used for imaging primary antibody staining. The bar is 5 µm and all images are at the same magnification.

**Figure 3:**
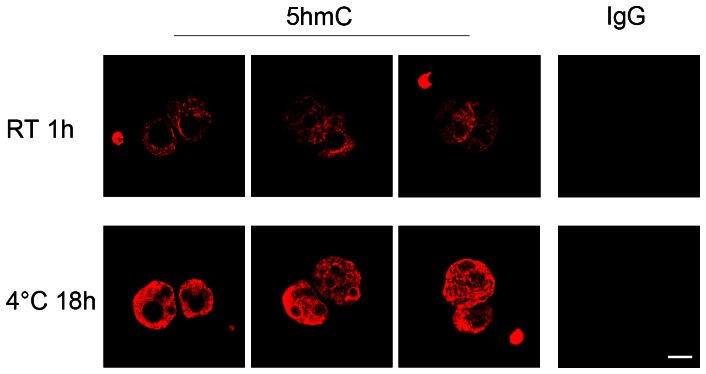
Staining of 5hmC with a monoclonal antibody. PN5 stage zygotes were collected directly from the oviduct and fixed and prepared for staining. They incubated with anti-5hmC or non-immune IgG for 1 h at room temperature (RT) or 18 h at 4°C after epitope retrieval by acid. Three representative images of each treatment are shown. These are representative of three independent replicates with more than 30 embryos per treatment in each replicate. The bar is 5 µm and all images are at the same magnification.

The minimum conditions required for solvent exposure of the 5meC and 5hmC epitopes in the zygote were next re-assessed. PN5 stage zygotes were collected directly from the oviduct and immediately fixed. Zygotes were either untreated or subjected to the two antigenic retrieval strategies (low pH to denature DNA alone, or followed by brief tryptic digestion to removal proteins that may bind to and mask the epitopes) (Fig. 2). Embryos were then exposed to anti-5meC for 18 h at 4°C or anti-5hmC at room temperature for 1 h. In the absence of either acid or trypsin treatment no 5meC signal was detected but a small amount of 5hmC signal was visible in both pronuclei. After acid treatment there was still little 5meC detected but a marked increase in 5hmC was observed. After further treatment by tryptic digestion a large increase in the level of 5meC staining in both pronuclei was observed but this did not result in any further detectable change in 5hmC (Fig. 2). These results show 5meC and 5hmC have different minimum requirements for solvent exposure within the PN5 stage zygote but that after both acid and trypsin treatment, both could be reliably detected. We have previously shown that the staining of 5meC in both pronuclei was evident when a range of different anti-5meC antibodies were used [21]. We show here that this was also the case for 5hmC. A monoclonal antibody to 5hmC also showed high levels of staining of both the male and female pronuclei in the zygote. This antibody showed similar thermodynamic requirements, as the polyclonal antibody. Incubation for 1 h at room temperature resulted in markedly lower levels of staining of both pronuclei than after incubation at 4°C for 18 h (Fig. 3). Under both conditions, staining of both the male and female pronuclei was evident.

**Figure 4:**
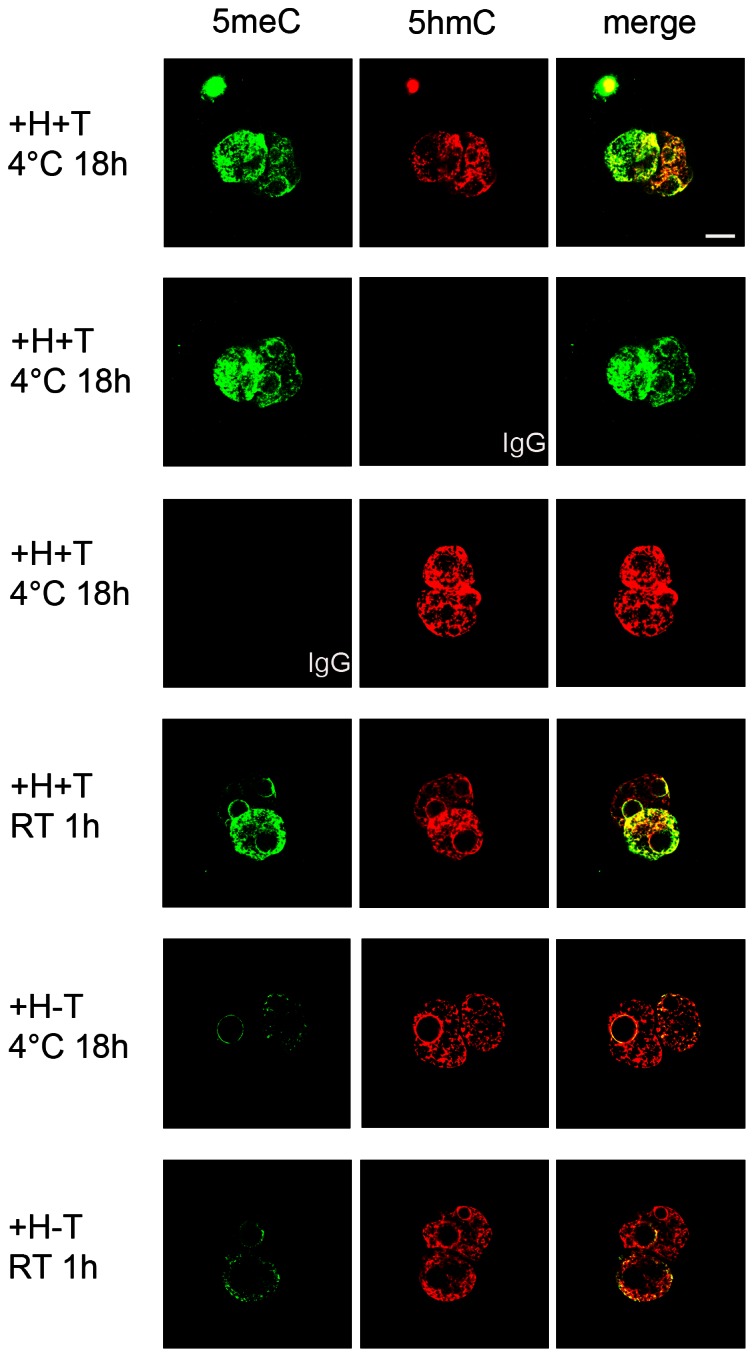
The effects of incubation time, temperature and epitope retrieval strategies on the simultaneous staining of 5meC and 5hmC. PN5 stage zygotes were collected directly from the oviduct and fixed and prepared for staining. They were co-incubated with anti-5meC and anti-5hmC for 1 h at RT or 18 h at 4°C after epitope retrieval by either acid and trypsin treatment (+H+T) or acid treatment alone (+H-T). 5meC was detected with an FITC-labeled secondary antibody (green) and 5hmC by a Alexa Fluor633 (red) label. The images are representative of at least three independent replicates. The signals from the green and red channels were merged to detect co-localization. The bar is 5 µm and all images are at the same magnification.

We next examined the effect of these parameters on the capacity to successfully co-stain 5meC and 5hmC in zygotes. PN5 stage zygotes were subjected to epitope retrieval by pre-treatment with acid followed by trypsin (+H+T) and then simultaneously stained with both anti-5meC and anti-5hmC. As controls, staining with one of the primary antibodies together with the corresponding non-immune immunoglobulin for the other primary antibody was performed. This analysis showed that after incubation with both antibodies for 18h at 4°C there was extensive co-localisation of both 5meC and 5hmC in both the male and female pronuclei. The pattern and intensity of staining was not markedly different when each single primary antibody was co-incubated with the corresponding non-immune IgG (Fig. 4). By contrast, when simultaneous co-staining was performed for only 1 h at room temperature staining for 5meC was relatively lower than 5hmC, particularly in the male pronuclei. 5meC was markedly reduced in both pronuclei compared to 5hmC, when staining (for 18 h at 4°C) was performed after acid denaturation as the only antigen retrieval step. The results confirm that the levels of these antigens detected in the zygote were remarkably sensitive to the staining conditions used. This demonstrates that staining under equilibrium conditions is an essential precondition to the use of this methodology for comparative analysis. High levels of co-localization of both antigens were present within each pronuclei of the PN5 stage zygote when equilibrium conditions were used.

**Figure 5:**
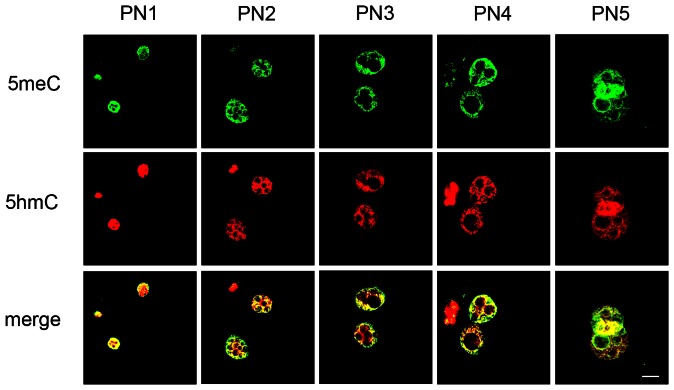
The patterns of 5meC and 5hmC staining in each pronuclei during zygotic maturation. Zygotes were collected directly from the oviduct at various times after fertilization and analyzed at the PN1-5 stages of development. Zygotes were subjected to antigen retrieval by both acid and trypsin treatment and were co-stained with anti-5meC and anti-5hmC for 18 h at 4°C. Anti-5meC was detected in the green channel and anti-5hmC in the red channel. The outputs from these channels were merged to show the areas of co-localization. For each primary antibody determined that provided no signal for the non-immune control immunoglobulin and these conditions used for detection of primary antibody. The same conditions for imaging were used for each stage of maturation. The results are representative of 3 independent replicates.

The patterns of co-staining throughout zygotic maturation were analysed using these validated conditions for equilibrium binding (Fig. 5). Zygotes were collected directly from the reproductive tract at intervals after ovulation and zygotes were examined at the PN1-5 stages of maturation. High levels of co-staining of both 5meC and 5hmC were observed at each of these stages of development in both the male and female pronuclei. At each stage of zygotic maturation some regionalised differences in the relative intensity of staining of 5meC and 5hmC was evident, with a tendency for 5meC to be more intense within the cortical regions of each pronuclei and 5hmC to be relatively more intense in the inner regions of the pronucleoplasm. As maturation progressed beyond the PN2 stage an increase in the structural complexity of the pronucleus was evident. This was most obvious in the male pronucleus, where a number of nucleolar precursor bodies (NPB) form. Staining of both 5meC and 5hmC was more intense in regions at the periphery of these emerging nucleoli. These changes were less advanced in the female pronucleus (Fig. 5).

**Figure 6:**
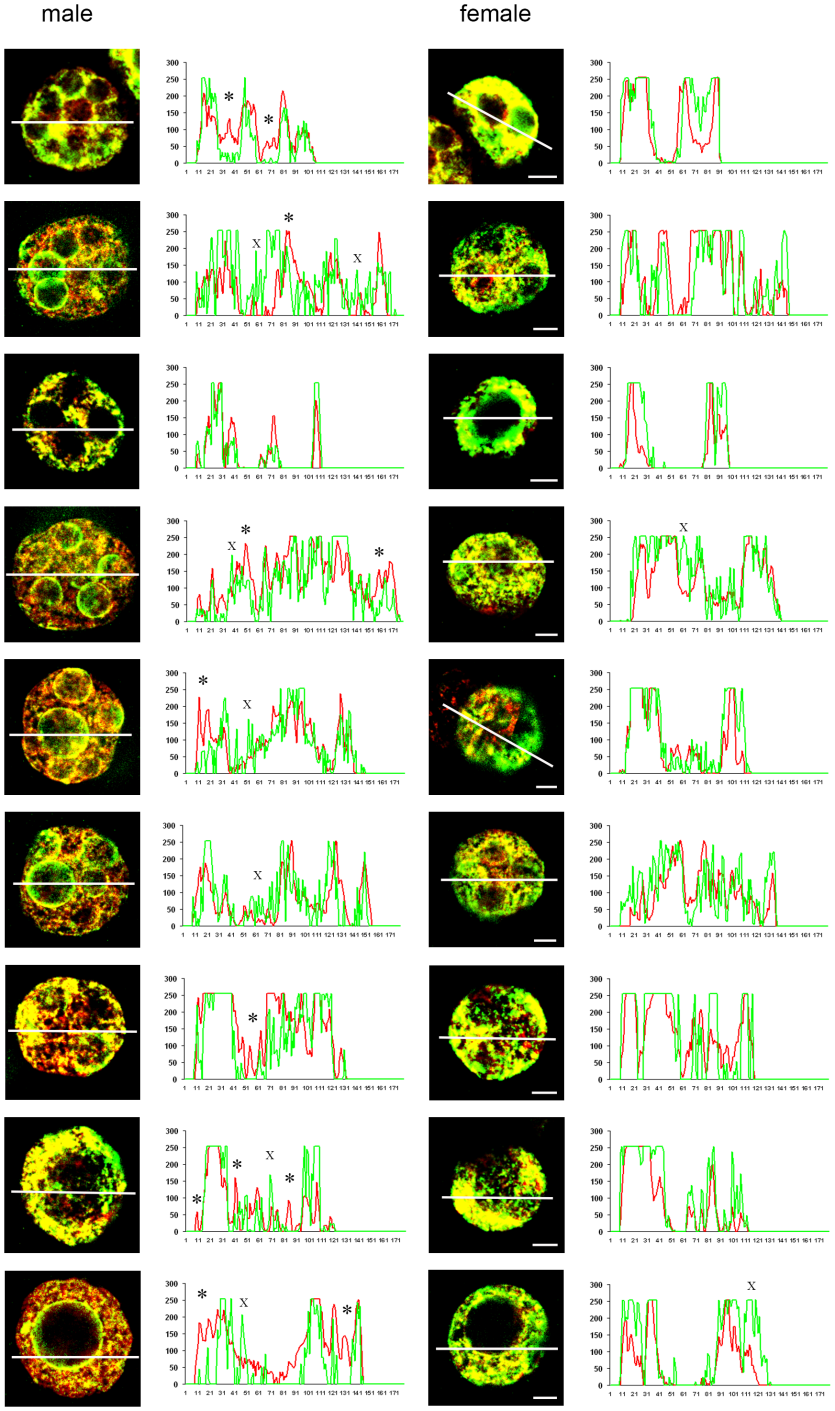
Analysis of heterogeneity in co-staining of 5meC and 5hmC in the male and female pronuclei of PN5 stage zygotes. Zygotes were collected directly from the oviduct and fixed and stained as in Fig. 4. Matched male and female pronuclei from individual zygotes are shown. Each image is a 1.03 µm equatorial cross-sectional image of each pronuclei. The image analysis program was then used to measure the optical density of staining of each antigen along the plane shown by the white line. Each corresponding graph shows the optical density per pixel (Y-axis, arbitrary units) along this plane axis, number of pixels). The green line on graphs shows intensity of 5meC staining and the red line is 5hmC staining along this path. Examples of areas where localized increases in 5meC staining was not accompanied by a similar magnitude increase in 5hmC staining are marked by (X) and accumulation of 5hmC relative to 5meC is marked by (*). Examples of nucleolar precursor bodies are shown by white arrows. The corresponding male and female pronuclei from each zygote were imaged and analyzed under identical conditions.

Fig. 6 shows a detailed analysis of these regionalised differences in staining of each antigen in the PN5 stage zygotes. Densitometric analyses are shown for matching male and female pronuclei from several zygotes. These analyses illustrate several features of staining: (1) there was considerable regional heterogeneity of both antigens across each the pronuclei; (2) in most regions the level of staining of 5meC and 5hmC changed with a similar pattern; (3) there were some regions where high levels of 5hmC occurred relative to 5meC (examples are marked * on figure), while other regions have elevated 5meC relative 5hmC (marked ^X^); (4) the most intense regions of staining, particularly of 5meC, occurred in the peri-nucleolar regions; (5) staining of both antigens was low within the NPB; and (6) there were more NPBs within the male pronuclei, accounting for the greater heterogeneity of staining within the male pronuclei.

## Discussion

This study defines the conditions required for the valid comparative analysis of 5meC and 5hmC in the mouse zygote. Each of these antigens had different levels of, and conditions for, solvent exposure in the zygote. While some 5hmC was solvent exposed when chromatin was in its native state after fixation, this was not the case for 5meC. The denaturation of chromatin achieved after brief acid treatment was sufficient to reveal most of the remaining detectable 5hmC but this treatment had limited capacity to expose 5meC in the PN5 zygote collected directly from the reproductive tract. By contrast, tryptic digestion was required to allow the detection of 5meC in both the male and female pronuclei, confirming our earlier results [21]. Using these methods of staining and antigen retrieval showed that both of these antigens were present in both pronuclei and these persisted throughout the processes of zygotic maturation. These results call for a reassessment of the current models of epigenetic reprogramming in the zygote. They are not consistent with a role for TET-mediated global demethylation in the zygote, and call into question the suitability of measurement of asymmetric staining of 5meC in the pronuclei of zygotes as a tool for the discovery and validation of putative mammalian demethylases.

The relative symmetry in levels of methylation staining of the male and female pronuclei are supported by bisulfite sequence analysis of a large number of CpG sites in the zygote (∼10^6^, <5% of total CpGs in the genome) [22]. Bisulfite analysis (and also HpaII/Msp1 restriction mapping), however, do not distinguish between 5meC and 5hmC (and other cytosine modifications) [24] and are therefore a measure of the net levels of all of the known cytosine modifications [25]. Consequently, bisulfite analysis alone can’t determine whether the observed symmetry of bisulfite measurements is a result of reciprocal conversion of 5meC to 5hmC in the male pronucleus [15], [16], [26]. The persistence of 5hmC in both pronuclei shown here and earlier [21] do not support a selective role for TET-mediated global remodelling of 5meC in the male pronucleus. It is possible that such remodelling may occur at the level of individual bases, but global analysis at the base level must await improved sensitivity of chemical analysis methods capable of discriminating between the various cytosine modifications in the very small amounts of DNA available from the zygote.

This study identifies the equilibrium conditions required for the valid use of widely used antibodies for analysis of 5meC and 5hmC in the embryo. It shows a similar outcome for two different anti-5hmC antibodies (one polyclonal and one monoclonal) and we have previously shown [21] that a range of antibodies against 5meC displayed similar patterns of staining as observed here. It is important to be mindful, however, that each new antibody source used should be subjected to its own individual validation.

While we found symmetry in the total level of 5meC and 5hmC staining, there was asymmetry in the structure of the male and female pronuclei, and this can create the illusion of asymmetric staining. Thus, the male pronucleus expands to a larger size than the female. This larger size means chromatin is dispersed through a larger volume, so staining of the 5meC and 5hmC is also dispersed over a large volume. Consequently, the level of staining intensity per unit cross-sectional area was reduced by this diluting effect, but the total staining across the entire equatorial cross-section of the pronuclei was not different. This illusion of staining asymmetry is further exacerbated by some asymmetry in the degree of structural heterogeneity within the male and female pronuclei. As zygotic maturation proceeded, the formation and number of nucleoli precursor bodies was most advanced within the male pronuclei. It is known that peri-centric heterochromatin forms rings around these nucleoli [17] and both 5meC and 5hmC accumulated in these regions. This finding is extended to show that under equilibrium antibody binding conditions the difference between male and female in the accumulation 5meC and 5hmC within these regions was still evident. The accumulation of 5meC tended to be more extensive than 5hmC, which meant that within the remaining nucleoplasm 5hmC was higher relative to 5meC.

This study shows that 5meC and 5hmC showed different minimum requirements for solvent exposure, but does not reveal the caused of this. The requirement for tryptic digestion to detect 5meC may infer that this antigen is masked by chromatin proteins in the late zygote. A range of well documented proteins that selectively bind 5meC have been described, but there is limited evidence for similar selective binding of proteins to 5hmC. MBD3/NURD may be one candidate, with evidence that these complexes may regulate expression by genes marked by 5hmC [27]. Most of the other documented methyl binding proteins have low affinity for 5hmC relative to 5meC [28]. The results of this study show that any proteins that may bind 5hmC in the zygote cause only limited steric interference with antibody binding, and this interference is readily displaced by the denaturation induced by acid treatment.

There is now a body of evidence that points to a need for reconsideration of the models of epigenetic reprogramming in the zygote. A number of species (sheep [29], [30], rabbit [31]) also do not show paternal global demethylation relative to the female pronuclei following fertilisation. It has also been shown that creation of mouse embryos from immature male germ cells is not accompanied by any observations of global loss of methylation, yet development can proceed normally [32]. The persistence of 5mC and 5hmC in both pronuclei throughout zygotic maturation indicate that this is not an optimal model for the search for a mammalian demethylase. It shows that an understanding of the role of 5hmC in the zygote and the early embryo is required. In this context it is noteworthy that there is a building body of evidence that 5hmC (and other cytosine modifications) may provide epigenetic information in their own right [25]. For example, 5hmC has an evolutionarily conserved role in marking promoter-distal and distal regulatory regions of the genome [25].

The high levels of 5meC and 5hmC observed throughout maturation of the normal mouse zygote point to a likely role for both these modifications in the epigenome of the early embryo. Evidence for the wide distribution of both antigens and differences in their behaviour during processing for immunolocalization suggests that each may provide different types of epigenetic information to the embryo. The unique characteristics of the zygotic genome provides for a powerful model for delineating the relative roles of each of these epigenetic modifications.

## Materials and Methods

### Animals

The use of animals was in accordance with the Australian Code of Practice for the Care and Use of Animals for Scientific Purposes and was specifically approved by the Royal North Shore Hospital Animal Care and Ethics Committee (Protocol number 0711-044). Hybrid (C57BL/6J X CBA/He) mice were used in all experiments. All animals were under 12 h light: 12 h dark cycle and had access to food and water ad libitum. Six week old females were superovulated by intraperitoneal injection of 5 IU equine chorionic gonadotrophin (Folligon, Intervet International, Boxmeer, The Netherlands) followed 48 h later by 5 IU human chorionic gonadotrophin (hCG, Chorulon, Intervet). Females were paired with males of proven fertility. Pregnancy was confirmed by the presence of a copulation plug the following morning (day 1).

### Mouse Embryo Collection

Zygotes were flushed from the oviduct with Hepes-buffered modified human tubal fluid medium (Hepes-mHTF) [33]. All components of the media were tissue culture grade (Sigma) and contained 3 mg bovine serum albumin per mL (Sigma). Zygotes were collected at various times after mating and staged according to the maturation of their pronuclei (PN1 being the least and PN5 the most mature stages of zygotic maturation prior to syngamy [34]). After collection, any surrounding cumulus cells were removed by brief exposure to hylauronidase (300 IU, Sigma). After washing in Dulbeccos phosphate buffered saline zygotes were immediately subjected to fixation.

### Immunolocalization Analysis

Immunofluorescence of zygotes was performed as previously described [21]. Embryos were fixed at room temperature in 4% (w/v) formaldehyde (prepared fresh daily from paraformaldehyde) for 30 min and then permeabilized in 0.5% (v/v) Tween20 and Triton100 at room temperature for 40 min. They were then blocked in 30% (v/v) heterologous serum at 4°C overnight followed by antigen retrieval methods. Unmasking of the 5meC antigen was performed by either brief exposure to 4N HCl for 10 min (to denature DNA) or a combination of acid denaturation and tryptic digestion (0.25% (w/v) trypsin at 37°C for 45 sec, Invitrogen, Carlsbad, CA). Digestion was stopped by washing in 10% serum. Tryptic digestion was performed to remove proteins that may potential mask the epitopes within DNA. Differing conditions for retrieval were used depending upon the experimental design and these are defined within the description of each experiment within the results section.

In this study we have used the two antibodies that are most widely used for detection of the 5meC and 5hmC antigens. Staining was performed by incubation with primary antibodies for periods of 1–18 h at either room temperature or 4°C, as described in individual experiments. The primary antibodies used are well described for the purpose: 5meC staining used mouse anti-5meC monoclonal antibody (Clone 33D3, MCA2201, AbD Serotec, UK ) and the secondary antibody was sheep anti-mouse IgG (FITC-labeled, 1∶300 dilution, Sigma); 5hmC staining used a rabbit anti-5hmC antibody (Cat#39769, Active Motif, USA) and the secondary antibody was Goat anti-rabbit IgG (1∶250 dilution, FITC-labeled, Sigma, or Alexa Fluor633, red, Molecular Probes, A21071). In one experiment an alternative mouse monoclonal anti-5hmC (ACTIVE MOTIF, Cat#39999) with the Alexa fluro 633 goat anti-mouse IgG 2a secondary antibody (Invitrogen, Cat#A-21136) was tested (all other experiments used the polyclonal anti-5hmC. In preliminary experiments the optimal concentration of each antibody was assessed to give saturable binding with the maximum signal to noise outputs under the conditions used. This analysis indicated use at concentrations of 1∶100 anti-5meC and 1∶150 (polyclonal) and 1∶500(monoclonal) anti-5hmC. DNA was counter-stained with propidium iodide (PI, 5 µg/ml) or DAPI and mounted with VECTASHIELD mounting medium (Vector Laboratories Inc, Burlingame, CA). Optical sectioning was performed with a Leica TCS SP5 confocal microscope equipped with 63x oil objective (1.4 NA, optical sections were 1.03 µm thick).

Zygotes from each treatment were processed at the same time and in parallel for each experimental replicate. All treatments were exposed to the same preparations and dilutions of all reagents including primary and secondary antibodies. Similarly, all preparations from an experiment were microscopically examined during the same session, and used identical microscope and camera settings. All image analysis was performed in an identical manner for all embryos within an experiment. For each experiment and replicate non-immune immunoglobulin control staining was performed as negative controls. Microscope and imaging settings were set so that no signal was detected from these negative controls.

Image analysis was performed with Image Pro-plus 6.3 (Media Cybernetics Inc, Rockville, MD). The outline of each pronuclei was defined by marking the regions stained by PI using the Area of Interest tool. The optical density of staining by primary and secondary antibodies combinations was then assessed within these areas of interest. Measurements included the average optical density (intensity)/pixel, the total staining (intensity by number of pixels within area of interest) and the cross-sectional area of the area of interest. Optical density along a define linear path through a section was performed using the Histogram function to indentify heterogeneity of staining within each pronuclei.
